# Junior Doctors' Enjoyment of Mental Health Placements in Derbyshire

**DOI:** 10.1192/bjo.2022.299

**Published:** 2022-06-20

**Authors:** Erum Shahid, Abigail Harlock, Abbas Ramji, Ritu Gupta, Vishnu Gopal

**Affiliations:** Derbyshire Healthcare NHS Foundation Trust, Derby, United Kingdom

## Abstract

**Aims:**

To assess the job and training satisfaction of junior doctors working in Mental Health placements in Derbyshire; to highlight areas of good practice and identify areas that need improvement to enhance their working experience.

**Methods:**

This is an ongoing Cycle of Quality Improvement to address Juniors Doctors enjoyment of work and job satisfaction. On a 25 point questionnaire we sought feedback as open response, graded response and free text. Questions were formulated using suggestions from Royal College of Psychiatrists Supported and Valued Review and BMA Fatigue and Facilities Charter. Advised areas of improvement from the previous 2017 Quality Improvement project were also reviewed and incorporated into the questionnaire design.

All junior trainees (including Core Psychiatry trainees, Foundation trainees, GP trainees and junior trust grade doctors) working between December 2020 to April 2021 in Derbyshire Healthcare NHS Foundation Trust were sent the questionnaire.

Official end of placement feedback from January-December 2020 was also compared to our findings.

**Results:**

15 doctors completed the questionnaire.

Areas of trainee-reported satisfaction included training on management of common psychiatric conditions (73%), weekly teaching sessions (100%), ability to organise leave (100%).

Areas of dissatisfaction included training on management of psychiatric emergencies (40%), poor regularity of supervision (53%), inadequate access to phlebotomy services (66%), ability to take adequate breaks (66%) and ability to fulfil training requirements (40%).

Discrepancies were noted in responses to similar questions in our questionnaire compared to the official end of placement feedback, with greater trainees answering with negative responses in this project.

**Conclusion:**

This project highlighted areas of high satisfaction for trainees and showed specific areas for improvement. Trainees responses have been reviewed with Educators and Trust Management for collaborative solutions, pilot schemes and future QI projects identified.

Observer bias was noted, with greater numbers of doctors answering similar questions negatively when feedback was anonymous, suggesting that they may be giving more honest answers when their identity is concealed.

